# Minimally invasive anterior muscle-sparing versus a transgluteal approach for hemiarthroplasty in femoral neck fractures-a prospective randomised controlled trial including 190 elderly patients

**DOI:** 10.1186/s12877-018-0898-9

**Published:** 2018-09-21

**Authors:** Franziska Saxer, Patrick Studer, Marcel Jakob, Norbert Suhm, Rachel Rosenthal, Salome Dell-Kuster, Werner Vach, Nicolas Bless

**Affiliations:** 1 Department of Orthopaedics and Traumatology, University Hospital Basel, Spitalstrasse 21, 4031 Basel, Switzerland; 2 Basel Institute for Clinical Epidemiology and Biostatistics, University Hospital Basel, Spitalstrasse 12, 4031 Basel, Switzerland; 3 Faculty of Medicine, University of Basel, Klingelbergstr. 61, 4056 Basel, Switzerland; 4 Clinic for Orthopaedics and Trauma Surgery Stephanshorn, Brauerstrasse 95, 9016 St. Gallen, Switzerland; 5 Department of Department of Anaesthesiology, Surgical Intensive Care, Prehospital Emergency Medicine and Pain Therapy, University Hospital Basel, Spitalstrasse 21, 4031 Basel, Switzerland

**Keywords:** Femoral neck fracture, Orthogeriatrics, Gerontotraumatology, Fracture hemiarthroplasty, Minimal invasive hemiarthroplasty, Trauma surgery in geriatric patients, Randomized controlled trial in the elderly

## Abstract

**Background:**

The relevance of femoral neck fractures (FNFs) increases with the ageing of numerous societies, injury-related decline is observed in many patients. Treatment strategies have evolved towards primary joint replacement, but the impact of different approaches remains a matter of debate. The aim of this trial was to evaluate the benefit of an anterior minimally-invasive (AMIS) compared to a lateral Hardinge (LAT) approach for hemiarthroplasty in these oftentimes frail patients.

**Methods:**

Four hundred thirty-nine patients were screened during the 44-months trial, aiming at the evaluation of 150 patients > 60 yrs. of age. Eligible patients were randomised using an online-tool with completely random assignment. As primary endpoint, early mobility, a predictor for long-term outcomes, was evaluated at 3 weeks via the “Timed up and go” test (TUG). Secondary endpoints included the Functional Independence Measure (FIM), pain, complications, one-year mobility and mortality.

**Results:**

A total of 190 patients were randomised; both groups were comparable at baseline, with a predominance for frailty-associated factors in the AMIS-group. At 3 weeks, 146 patients were assessed for the primary outcome. There was a reduction in the median duration of TUG performance of 21.5% (CI [− 41.2,4.7], *p* = 0.104) in the AMIS-arm (i.e., improved mobility). This reduction was more pronounced in patients with signs of frailty or cognitive impairment. FIM scores increased on average by 6.7 points (CI [0.5–12.8], *p* = 0.037), pain measured on a 10-point visual analogue scale decreased on average by 0.7 points (CI: [− 1.4,0.0], *p* = 0.064). The requirement for blood transfusion was lower in the AMIS- group, the rate of complications comparable, with a higher rate of soft tissue complications in the LAT-group. The mortality was higher in the AMIS-group.

**Conclusion:**

These results, similar to previous reports, support the concept that in elderly patients at risk of frailty, the AMIS approach for hemiarthroplasty can be beneficial, since early mobilisation and pain reduction potentially reduce deconditioning, morbidity and loss of independence. The results are, however, influenced by a plethora of factors. Only improvements in every aspect of the therapeutic chain can lead to optimisation of treatment and improve outcomes in this growing patient population.

**Trial registration:**

www.clinicaltrials.gov: NCT01408693 (registered August 3rd 2011).

**Electronic supplementary material:**

The online version of this article (10.1186/s12877-018-0898-9) contains supplementary material, which is available to authorized users.

## Background

Femoral neck fractures (FNFs) are typically associated with old age and represent a major cause of morbidity, functional dependence and socio-economic burden. These fractures also represent a sign of frailty, associated with a one-year mortality of approximately 30% [1–3] The affected patient population is very heterogeneous [4–6], with pre-fracture characteristics like age, the number of independent activities of daily living (ADLs) or the presence of dementia [6] being prognostic factors for long-term outcome [4].

**Fig. 1:**
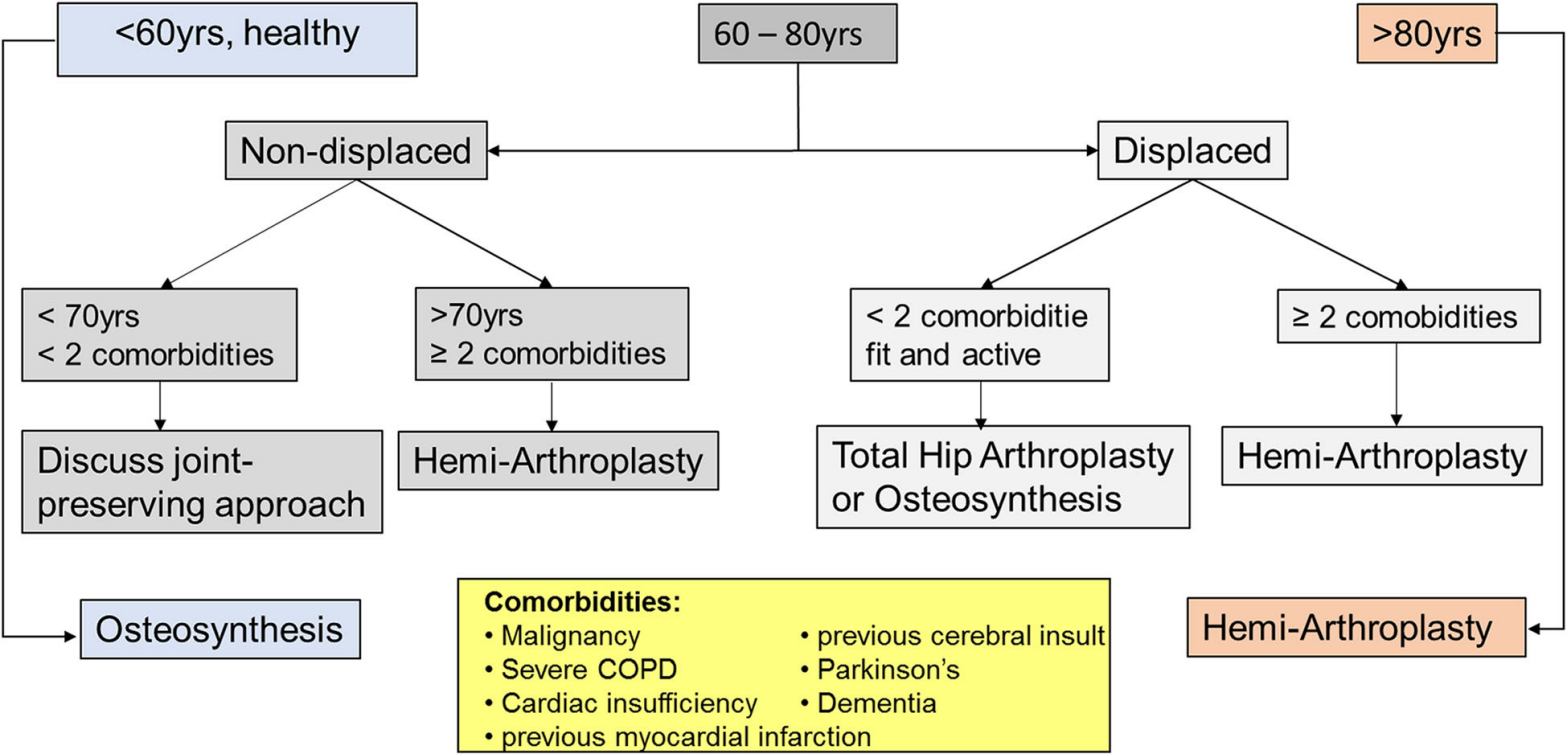
Institutional treatment algorithm for femoral neck fractures. Algorithm for the treatment of FNF with a high degree of personalizability according to the specific patient characteristics, with a focus is on early pain free and fully weight-bearing mobilization

The treatment of FNFs is a matter of debate and varies with age, fracture pattern and general health [7, 8]. For elderly patients with FNFs, conservative treatment and osteosynthesis have mostly been abandoned [8–12]. At our institution, many elderly patients are treated with cemented [13] hemiarthroplasty (HA) due to better short-term results, avoidance of suffering, efficient restoration of functional capacities and preservation of independence [9–12, 14, 15] (see Fig. 1 for the institutional treatment algorithm).

Approaches for joint replacement though can differ considerably in their invasiveness [16]. The lateral transgluteal Hardinge (LAT) approach [17] has been one of the standard approaches for joint replacement of the hip for decades. In the twenty-first century, the anterior minimally invasive Hueter (AMIS) approach regained popularity [18–20]. This approach allows a muscle-sparing access to the hip joint using an inter-muscular and inter-nervous plane, while the transgluteal approach necessitates the transection of muscular tissue. The rationale for using the AMIS approach is a potential reduction of soft tissue damage, blood loss and postoperative pain, as well as an acceleration of postoperative mobilisation, with a consecutive reduction of length of stay (LOS) and duration of rehabilitation [21–25] compared to other approaches.

Despite several publications, there is no consensus on the optimal approach for total hip arthroplasty (THA) [26, 27]. Furthermore, the reported data after THA have typically been derived from a younger and healthier population of patients with osteoarthritis [21–24]. Evidence from geriatric patients suffering FNFs is scarce [28–32].

Therefore, the primary objective of the trial was the comparison of a minimally invasive to a more conventional approach for the implantation of cemented HA in elderly patients suffering from FNFs. The “Timed up and go” test (TUG) [33] at 3 weeks was chosen as the primary endpoint due to its prognostic value for long-term mobility and independence [34, 35]. A reduction of 20% in the TUG duration was assumed to be clinically relevant [36]. The secondary objectives of the trial were an evaluation of the changes in functional independence and TUG performance during the first postoperative year; the description of differences in the surgical performance, complications and mortality between the two approaches; and a subgroup analysis on the influence of functional independence and cognitive impairment.

## Methods

The trial was designed as a prospective single-centre randomised controlled trial (RCT) with a one-year follow-up period at a level-one orthopaedic and trauma centre. From 09/11 to 04/15, we screened all consecutive, previously ambulatory patients 60 years or older with an FNF eligible for HA according to our institutional algorithm (Fig. 1). For inclusion and exclusion criteria, see Table 1.

**Table 1:** In- and Exclusion Criteria

Inclusion Criteria	Exclusion Criteria
Age of 60 years or more, ambulatory with/without walking aid before trauma	Multiple fractures
Femoral neck fracture eligible for hemi-arthroplasty in accordance with the algorithm for femoral neck fractures	Suspicion of a pathological fracture in the context of known or unknown malignancy
	Previous surgery on the injured femur
Informed consent in surgery and trial- participation	Refusal of trial participation by the patient or legal representatives

In- and exclusion criteria chosen deliberately broad to increase the external validity of the results. The exclusion criteria based on the pattern of injury were defined to avoid an interference with early mobility by e.g. the inability to use walking aids due to fractures of the upper extremity, or lesions to the contralateral leg

Eligible patients entered the informed consent process. Depending on their cognitive abilities (quantified using a mental status questionnaire [37], MSQ), they were individually or in the presence of a designated proxy informed about the diagnosis, the proposed treatment and the randomised trial. The surgeon on call included patients only if they consented in writing or – in case of relevant cognitive impairment – gave their verbal assent with written consent by a designated proxy according to Swiss civil code (Art. 378) or a legal guardian. The trial was approved by the ethics committee (Ethikkommission beider Basel) EKBB Reference No. 68/11) and followed good clinical practice, as well as the Declaration of Helsinki. The trial has been registered with clinicaltrials.gov, and the complete protocol is available as additional files.

After consent to surgery and trial participation, the surgeon on call randomised the patients using a web-based randomisation tool (randomizer.at) with the “Completely At Random” option. The randomised treatment was delivered as soon as possible (i.e., 31% of patients underwent operation on the day of randomisation, 48% the next day, and only 2% of the patients had to wait 2 or more days for surgery, including those waiting for medical reasons like oral anticoagulation, etc).

At baseline, the following patient characteristics were recorded: age, gender, body mass index (BMI), residential status, use of a walking aid, diagnosis of dementia, number of medications (substances), Charlson Comorbidity Index [38], and ASA (American Society of Anesthesiologists) grading [39]. Additionally, standard laboratory parameters were recorded. The pre-fracture Functional Independence Measure (pfFIM) [40] status was assessed retrospectively.

Surgery was performed after standard perioperative antimicrobial prophylaxis. Both groups were operated on using a cemented AMIS© stem (Medacta International, Castel San Pietro, Switzerland) and a monopolar head (Mathys AG, Bettlach, Switzerland) according to the manufacturer’s instructions (including the use of monopolar heads by another manufacturer, since initially monopolar heads were not available in the shaft manufacturer’s product range) and our standard operating procedures.

For the LAT approach, an incision of 8–14 cm is centred on the trochanter major to expose the fascia latae. After splitting this structure, the vasto-gluteal sling is exposed (the complex of the gluteus medius muscle proximally, the trochanter major and the vastus lateralis muscle distally). For the preparation of the capsule, the muscles are split in line with their fibres in the anterior third of the muscular body forming a musculo-tendineous flap.

For the AMIS approach, a 7- to 10-cm incision is made 2 cm latero-distally to the antero-superior iliac spine towards the fibular head. The interval between the sartorius muscle medially and the tensor fascia latae laterally is developed. The capsule is exposed between the rectus femoris muscle medially and the vastus intermedius muscle laterally.

All patients underwent the same postoperative aftercare with thrombo-embolic prophylaxis as well as mobilisation with full weight bearing under physiotherapeutic guidance from day one, using standard analgesia. As part of orthogeriatric care [41], patients were followed-up and treated for osteoporosis, fall prophylaxis, malnutrition and delirium. The latter was assessed by the nursing staff using a modified Delirium Observation Screening scale (DOS) [42] and – in case of pathology – the Confusion Assessment Method (CAM) [43].

Intraoperative data, such as the duration of surgery or intraoperative blood loss, were recorded. The latter was assessed via the number of erythrocyte concentrates ordered perioperatively up to 72 h post-surgery. Perioperative in-hospital complications were assessed as secondary outcome variables using the Clavien-Dindo classification [44] to differentiate general and surgery-related complications. After hospitalisation, only the occurrence of serious adverse events (SAE), surgery-related complications and implant-related infections were recorded. LOS was documented, as was the discharge destination (in-patient rehabilitation, nursing home, etc.). After discharge, follow-up was scheduled for 3 (only functional assessment), 6 and 12 weeks and one year postoperatively for clinical examination, functional assessment and X-rays as per the institutional standard.

The first assessment of functional recovery was performed in-hospital on day 5. At every follow-up TUG [33, 45], FIM [40] and pain scores were recorded by an independent study nurse blinded to the treatment allocation. TUG is an assessment of physical mobility validated in elderly patients with high discriminative potential at early time points [46] and a predictive value for the long-term functional outcome [34, 35]. The test measures the time needed by an individual to get up from a chair (seat height 45 cm, arm height 65 cm), walk a distance of 3 m with habitual shoes and a walking aid, and sit down again [33]. The FIM [40] is an assessment of ADL that evaluates 13 motor and 5 cognitive faculties. Pain was assessed using a 0- to 10-point visual analogue scale (VAS).

The literature describes unfavourable outcomes in patients affected by frailty. The definition of frailty remains controversial [47]. Frailty often is described as a multidimensional dynamic state of increased vulnerability and loss of resistance to external stressors, resulting in an increased risk of adverse outcomes [48]. The protocol envisaged a subgroup analysis based on the MSQ scores as a surrogate marker for frailty. This was complemented by visualising treatment effects in dependence on pfFIM status, given the inverse relationship between the baseline FIM and frailty [49].

At the time of submission, the authors became aware of a publication by Arjunan et al. [50], which described a frailty index based on the FIM, medication count and comorbidities, aspects that had all been assessed during the present trial. Therefore, an additional post hoc subgroup analysis based on the described frailty index was performed. The computation of this frailty index is outlined in the Additional file 1.

The sample size calculation used the original description of the test [33] to obtain information on the distribution to be expected for the primary outcome. This resulted in a sample size of 150 patients to be included in the analysis in order to demonstrate a percentage difference in medians of 20% (corresponding to 6 s) with 80% power. Assuming a drop-out rate of 20% after experience from trials in the same patient population, an inclusion of 190 patients was planned. A recent investigation on the minimum clinical important difference (MCID) of the TUG [36] identified an MCID of 6 s when using quality of life assessed by the EQ-5D as an anchor and the minimum detectable change approach, and even smaller differences when using other anchors and approaches.

### Statistical analysis

The distribution of continuous variables is described by mean/SD or selected percentiles as appropriate. The distribution of ordinal and binary variables is described by absolute and relative frequencies. The significance of differences between the groups was assessed with the Wilcoxon rank sum test for continuous and ordinal variables and with Fisher’s exact test for binary variables.

The outcome “duration of TUG performance” was compared between the two treatment arms using a regression model for the log-transformed values with adjustment for pfFIM status and age, allowing us to estimate a percentage difference in medians (details are given in the Additional file 2 and Additional file 3: Figure S1). Other continuous outcomes including VAS scores were analysed with a linear regression model, such that effects (delta) correspond to mean differences. Binary outcomes were analysed with a generalised linear model with log link and binomial outcome distribution reporting effects as relative risks. The one-year mortality is presented as percentages derived from a Kaplan-Meier-estimate, but effects are described as hazard ratios based on a Cox regression model. Treatment arms were defined according to randomisation, and adjustments were performed using the same covariates as in analysing the primary endpoint. The distribution of continuous outcomes was visualised in relation to treatment arm and pre-fracture FIM status by scatter plots mimicking the adjustment for this factor in the analyses. Boxplots were used in subgroup analyses, and the distribution of ordinal outcomes was visualised by pie charts.

Patients who declined to perform the TUG were excluded from the analysis; those who could not start or failed to complete the test were included in the analysis with a value of 300 s, which lies distinctly above the maximally observed value. To increase the rate of follow-up, patients who declined follow-up at our institution were offered an assessment at their location of residence.

TUG at 3 weeks postoperation was defined as the primary outcome in the protocol, with a list of secondary outcomes like LOS, intraoperative aspects, the performance of activities of daily living (FIM), mortality, etc. We added pain, implant-related infections and the return to pre-fracture ambulatory status (with or without a walking aid) after 3 and 12 months. The protocol furthermore suggested a subgroup analysis within the patient groups defined by MSQ scores ≤7 and MSQ scores ≥8 corresponding to abnormal vs normal values, respectively [37].

All computations were performed using Stata 14.2 (StataCorp. 2015. College Station, TX: StataCorp LP). A significance level of 5% was used.

## Results

### Patient flow and characteristics

**Fig. 2:**
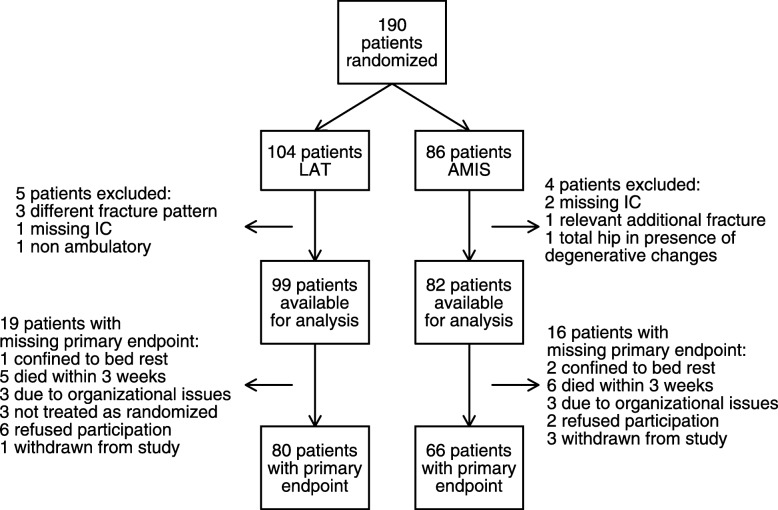
Flow of patients in the two trial arms. Flowchart documenting the reasons for unavailability for analysis after randomization

Over 44 months (September 1st, 2011 to April 15th, 2015), 448 patients were screened for inclusion, 258 were not eligible (see Table 2), and 190 were randomised. The primary endpoint was assessed in 146 patients (for details, see Fig. 2). Participation in follow-up visits and the availability of TUG and FIM scores are depicted in Additional file 4: Table S1. The participation rate declined over time in both arms, and 75% attended the 3-month follow-up visit. Conversely, on day 5, 20% did not perform the TUG, and later the rate of TUG performance rose to 95%. Except for day 5, when TUG was more readily performed by LAT patients, the rates were similar between the two treatment arms. FIM scores were missing in 13 patients at baseline (equally distributed between the treatment arms) and 6 patients at day 5 (only from the AMIS-arm). Investigations regarding predictors for drop-out are displayed in the Additional file 5 and Additional file 6: Table S2.

**Table 2:** Reasons for non-inclusion

Exclusion Criterion	n total 258
Total hip arthroplasty	53
Joint preserving strategy	5
Additional or other fracture	33
Non-ambulatory on admission	23
Underlying malignancy or neurologic disease	22
Death before inclusion	3
Refusal of surgery	7
Intercurrent contralateral femoral fracture	12
Patients’ refusal of consent to trial	37
Guardians’/proxies’ refusal of consent to trial	36
Unclear IC situation (demented but no guardian or family etc.)	10
Logistics (tourists, commuters not insured in Switzerland etc.)	17

The reasons for non-inclusion mirror the heterogeneity of the patient population. While some patients are active and un-burdened by comorbidity with the indication for total hip arthroplasty as personalized treatment strategy, others are non-ambulatory on admission. Also legitimate informed consent is a sensitive topic. In unclear situations, patients had to be excluded. All other patients either gave consent or proxy consent was obtained with patients’ assent. Only 12 patients suffered an intercurrent femoral fracture which may be explained by the evaluation of all patients suffering FNF by a fracture liaison service that established basic prophylaxis, diagnostics and treatment for osteoporosis [35]

Table 3 shows the baseline patient characteristics in the two study arms. The patients were on average 84.2 years old and predominantly female. It should be noted that in spite of randomisation, there was a certain imbalance between the groups. The AMIS-arm had slightly more female patients (77% vs 66%), as well as a higher level of support in the living situation (65% vs 43%), more frequent use of walking aids (63% vs 46%) and a higher rate of dementia (32% vs 20%). However, both groups were comparable with respect to BMI, MSQ score [37], pfFIM status, number of medications, Charlson Comorbidity Index [51], ASA score [52], time until surgery and laboratory parameters.

**Table 3:** Baseline characteristics in the two treatment-arms

	LAT	AMIS	
Age			
N	99	82	*p* = 0.767
Mean (sd)	84.0 (6.6)	84.4 (6.7)	
Median (10%, 90%)	84.0 (75.0,92.0)	86.0 (74.0,92.0)	
Gender			
Male	33/99 33.3%	19/82 23.2%	*p* = 0.134
Female	66/99 66.7%	63/82 76.8%	
Body Mass Index			
N	94	72	*p* = 0.689
Mean (sd)	23.7 (4.9)	23.8 (4.8)	
Median (10%, 90%)	24.0 (18.0,29.0)	23.0 (18.0,29.0)	
Residential status			
Own home	56/98 57.1%	37/82 45.1%	*p* = 0.127
Own home supported	15/98 15.3%	12/82 14.6%	
Assisted living	4/98 4.1%	9/82 11.0%	
Nursing home	20/98 20.4%	23/82 28.0%	
Other	3/98 3.1%	1/82 1.2%	
Walking aid			
Yes	45/98 45.9%	51/81 63.0%	*p* = 0.023
Dementia			
Yes	20/98 20.4%	26/82 31.7%	*p* = 0.084
MSQ			
N	92	77	*p* = 0.312
Mean (sd)	7.9 (2.9)	7.5 (3.1)	
Median (10%, 90%)	9.0 (3.0,10.0)	9.0 (2.0,10.0)	
pfFIM			
N	93	75	*p* = 0.860
Mean (sd)	107.0 (26.6)	107.8 (24.5)	
Median (10%, 90%)	123.0 (63.0,126.0)	120.0 (67.0,126.0)	
Number of medications			
N	97	82	*p* = 0.524
Mean (sd)	6.9 (4.4)	6.5 (4.4)	
Median (10%, 90%)	6.0 (2.0,13.0)	6.0 (1.0,12.0)	
Charlson Comorbidity Score			
N	98	79	*p* = 0.952
Mean (sd)	2.3 (2.1)	2.3 (2.2)	
Median (10%, 90%)	2.0 (0.0,5.0)	2.0 (0.0,5.0)	
Frailty Index			
N	92	73	*p* = 0.739
Mean (sd)	0.18 (0.15)	0.17 (0.16)	
Median (10%, 90%)	0.12 (0.01,0.40)	0.13 (0.03,0.41)	
ASA score			
2	33/99 33.3%	23/82 28.0%	*p* = 0.436
3	62/99 62.6%	55/82 67.1%	
4	4/99 4.0%	4/82 4.9%	
Time until surgery (hours)			
N	97	82	*p* = 0.943
Mean (sd)	27.4 (18.9)	25.6 (14.4)	
Median (10%, 90%)	23.0 (7.0,52.0)	24.0 (7.0,44.0)	
Haemoglobin			
N	98	82	*p* = 0.901
Mean (sd)	129.5 (16.1)	129.9 (15.7)	
Median (10%, 90%)	132.5 (109.0,149.0)	130.0 (107.0,149.0)	
Creatinine			
N	95	76	*p* = 0.596
Mean (sd)	19.8 (10.1)	19.5 (11.9)	
Median (10%, 90%)	17.0 (10.0,30.0)	17.0 (10.0,29.0)	
Albumin			
N	98	82	*p* = 0.674
Mean (sd)	33.7 (6.9)	34.0 (4.7)	
Median (10%, 90%)	35.0 (28.0,40.0)	34.0 (29.0,39.0)	
CRP			
N	98	82	*p* = 0.512
Mean (sd)	15.7 (29.7)	17.9 (27.1)	
Median (10%, 90%)	4.0 (1.0,58.0)	6.0 (0.0,49.0)	
Leukocytes			
N	98	82	*p* = 0.997
Mean (sd)	10.3 (3.7)	10.2 (3.6)	
Median (10%, 90%)	10.0 (6.0,15.0)	10.0 (6.0,15.0)	

Basic patient characteristics, although the groups are comparable there is a higher degree of frailty associated factors apparent in the AMIS- arm with a higher level of support (65% vs 43%), a more frequent use of walking aids (63% vs 46%) and a higher rate of diagnosed dementia (32% vs 20%)

Over time, 38 different surgeons performed 179 operations. Five surgeons performed 13 or more operations, and 19 surgeons operated 2–8 times. All surgeons were board-certified but had varying levels of experience. The surgeon on duty, who was always supervised by a senior surgeon experienced in both approaches, performed the intervention.

### Primary endpoint

One-hundred and twenty-six patients (86%) performed the TUG at the 3-week follow-up with a duration of TUG performance between 11 and 266 s (median 30 s, mean 46 s). Twenty patients (12 LAT, 8 AMIS) could not start or finish the test and entered the analysis with a value set to 300 s:

- 2 patients had too poor of a performance status to start the test;
- 2 patients suffered from too much pain to start the test;
- 2 patients could not move without assistance;
- 5 patients could not move to a chair;
- 7 patients could not stand up from a chair; and
- 2 patients could not walk 3 m.

The analysis of the primary outcome, i.e., the duration of TUG, was adjusted for pfFIM status and age (cf. the Additional file 2). For two patients, pfFIM status was not available, so 144 patients remained in the adjusted analysis. Additional file 7: Table S3 shows the distribution of baseline characteristics among the patients entering this analysis. Compared to Table 3, the imbalance of some characteristics – in particular pfFIM – was more pronounced in this adjusted analysis.

**Fig. 3:**
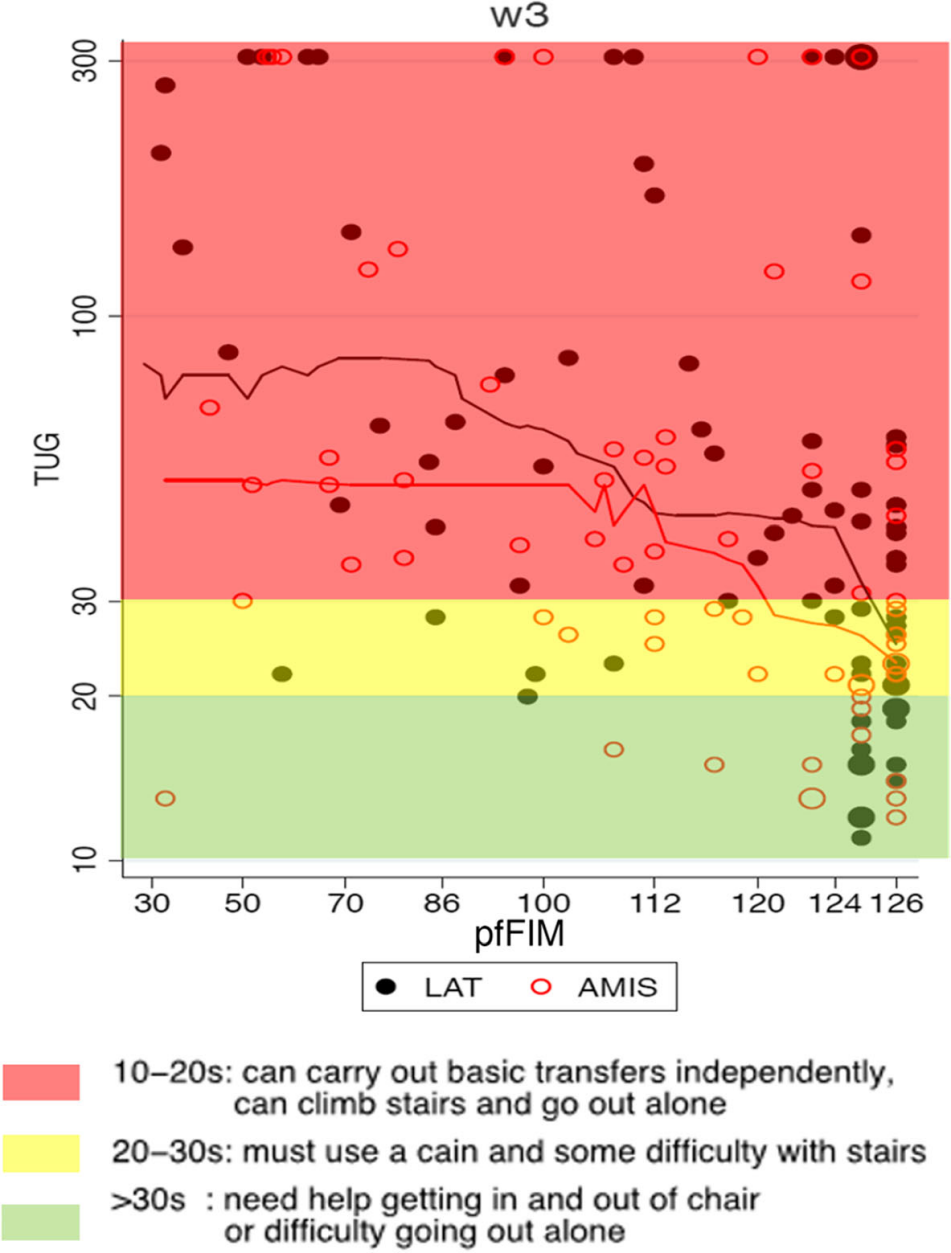
The distribution of duration of TUG performance (DTP) at the 3 weeks follow up visit in relation to treatment arm and pfFIM. Visualisation of the duration of TUG performance in the context of its clinical relevance. The green background signifies independence, while the yellow and red imply an increasing degree of dependence for mobilisation (yellow) and basic activities of daily living (red)

The distribution of the primary endpoint is visualised in Fig. 3. The percentage difference in median was estimated as a 21.5% shorter duration of TUG performance (i.e., − 21.5%) favouring the AMIS-arm with a 95% CI of [− 41.2, 4.7] and a *p*-value of 0.101. Analysing the same patients without adjustment for baseline variables resulted in a percentage difference of − 17.2% (CI [− 39.8, 14.0], *p* = 0.249). Investigations concerning the sensitivity of these results to the handling of missing values in the primary outcome and the pfFIM status are elaborated in the Additional file 8 and Additional file 9: Table S4. Figure 3 suggests a more pronounced treatment effect in patients with low pfFIM status.

### Secondary outcomes

**Fig. 4:**
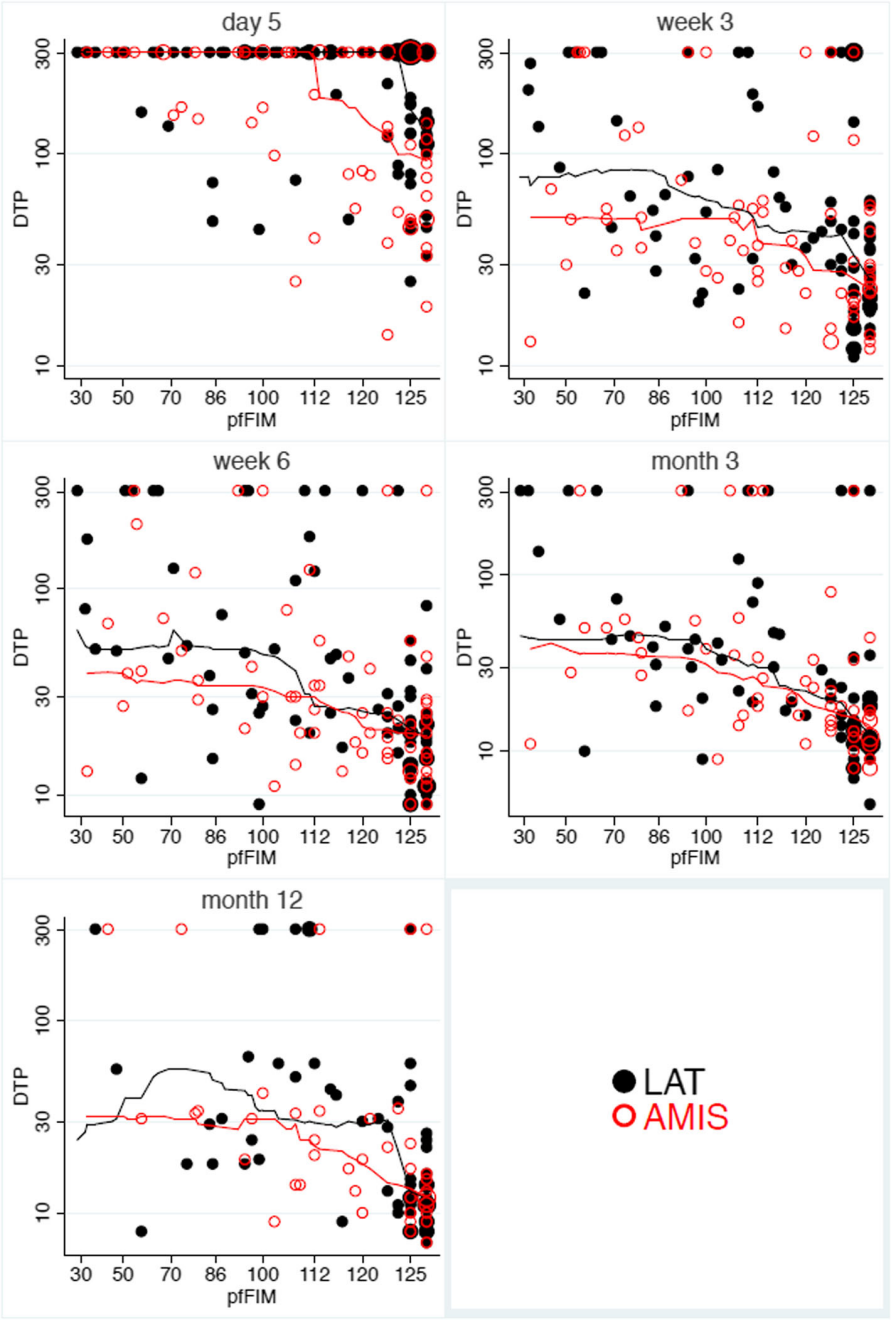
Distribution of duration of TUG performance (DTP) at day 5 and at all follow up visits in relation to treatment and pfFIM. Individual values for patients’ TUG performance in relation to their pfFIM and treatment. The running median curves are based on the next 25 neighbours on both sides of an observation and illustrate larger differences in patients with lower pfFIM. Note that the y-axis uses a logarithmic scale

Figure 4 shows the distribution of the duration of TUG performance at all time points in relation to treatment arm and pfFIM status. The median duration was lower in the AMIS-arm at all time points, with a decreasing treatment difference over time (cf. Table 4). The advantage for patients with low pfFIM status is more pronounced at earlier time points.

**Table 4:** Treatment effects on duration of TUG performance (DTP), FIM and pain over time

	LAT	AMIS	effect	95% CI	*p*-value
	*n*	*n*			
DTP					
Day 5	83	62	−24.4	[−40.4, −4.2]	0.022
Week 3	79	65	−21.5	[−41.2, 4.7]	0.101
Week 6	80	63	−16.4	[−36.9, 10.8]	0.216
Month 3	75	58	−10.1	[−34.9, 24.2]	0.520
Month 12	55	42	−10.3	[−40.3, 34.9]	0.604
FIM					
Day 5	93	75	3.9	[−1.8, 9.7]	0.181
Week 3	89	72	6.7	[0.5, 12.8]	0.037
Week 6	84	66	5.5	[−1.1, 12.2]	0.106
Month 3	77	60	3.6	[−4.1, 11.4]	0.361
Month 12	59	47	3.6	[−5.2, 12.4]	0.427
VAS					
Day 5	68	51	−0.8	[−1.5,-0.1]	0.026
Week 3	86	70	−0.7	[−1.4,0.0]	0.064
Week 6	81	63	−0.2	[− 0.8,0.3]	0.433
Month 3	76	59	−0.4	[−0.8,0.0]	0.075
Month 12	59	47	−0.0	[−0.5,0.4]	0.935
Back to pfFIM-level:					
Day 5	93	75	1.1	2.7	0.587
Week 3	89	72	4.5	6.9	0.515
Week 6	84	66	7.1	16.7	0.076
Month 3	77	60	33.8	26.7	0.456
Month 12	59	47	49.2	38.3	0.326

Treatment effects during the first postoperative year

Upper three panels: Effect-estimates with 95% confidence intervals and *p*-values for the outcomes DTP, FIM and pain at each time point. The effect for DTP is expressed as the percentage difference in median. The effect for FIM is the difference in the mean change from baseline. The effect for pain is the difference in mean VAS score. The effects refer to the difference AMIS minus LAT

Lower panel: Percentage of patients with FIM-values equal or above their pre-fracture values

Patients in the AMIS-arm had higher FIM values at almost all time points during the one-year follow-up after baseline, in particular for patients with low pfFIM status (see Additional file 10: Figure S2). The corresponding effect estimates in Table 4 suggest that the effect on the FIM was the largest at week 3 (difference in mean values 6.7 points, CI:[0.5,12.8], *p* = 0.037), but to some degree, the effect was still present after 12 months (3.6, CI: [− 5.2,12.4], *p* = 0.427). During the first postoperative year, a substantial fraction of patients reached FIM levels comparable to their pfFIM status. This proportion at early time points was higher in the AMIS-arm, and the results were reversed at later time points (see Table 4). The complete individual trajectories for the duration of TUG performance and FIM scores are shown in Additional file 11: Figure S3.

AMIS patients suffered less pain at all time points, in particular those with low pfFIM values. The distribution of the VAS scores in relation to treatment and pfFIM status is detailed in Additional file 12: Figure S4. The corresponding effect estimates in Table 4 indicate a clinically relevant advantage, especially in the early postoperative period, when taking into account that the mean VAS score in the whole population was only 1.6 at day 5 and decreased thereafter.

The difference between AMIS and LAT with respect to other outcome variables is depicted in Table 5, indicating a lower degree of postoperative delirium, a shorter LOS, a lower need for blood transfusions, and a shorter operative time for the AMIS-arm. Infections and death were more frequent after AMIS, but SAEs during follow-up occurred less frequently. The chance of returning to “No walking aid” was similar in both arms. The one-year mortality was 20% in the LAT-arm and 28% in the AMIS-arm (adj. HR 1.64, CI: [0.84,3.21], *p* = 0.149). Five patients died following implant-related infections, 3 of these from the LAT and 2 from the AMIS-arm, and all cases followed the patients’ wishes for best supportive care.

**Table 5:** Differences in secondary outcomes between the two treatment groups

							Adjusted	
	LAT	AMIS	LAT	AMIS	Effect	*p*	Effect	*p*
	*n*	*n*	Mean	Mean	Delta		Delta	
Postoperative delirium	89	77	0.94	0.87	−0.07	0.667	−0.12	0.468
LOS	96	78	11.39	10.97	−0.41	0.630	−0.70	0.393
Operative time	97	82	100.1	96.3	−3.8	0.419	−5.3	0.253
Erythrocyte concentrates within 72 h	97	82	0.73	0.50	−0.23	0.174	−0.31	0.078
			%	%	RR		RR	
Implant related infections	96	79	5.2	8.9	1.70	0.347	1.81	0.281
SAE during follow up	90	73	30.0	26.0	0.87	0.577	0.90	0.682
Return to no WA at 3 months	45	23	28.9	26.1	0.90	0.809	0.80	0.578
Return to no WA at 12 months	34	17	61.8	64.7	1.05	0.836	0.94	0.829
			%dead	%dead	HR		HR	
1 year mortality	99	82	20.2	28.0	1.46	0.205	1.64	0.149

The results are more favorable in the LAT arm for the avoidance of implant related infections and the one-year mortality. There is also an advantage for return to no walking aids at three months. The AMIS arm, on the other hand was more favorable for all the other aspects analyzed. However, none of the differences reached statistical significance. Adjustment was performed for pfFIM and age

*WA* Walking aid, *SAE* serious adverse event or surgery related complication

In the LAT-arm, 5 patients showed wound healing problems, and 46 had documented significant postoperative haematoma. Five patients in the LAT-arm developed an implant-related infection. Seven patients from the AMIS-arm had implant-related infections. All of these cases were associated with soft tissue complications like haematoma or wound healing disturbance. In the AMIS-arm, no other patients with soft tissue complications were noted. All infections were diagnosed during the first 6 postoperative weeks. Treatment followed standard practice [53]. Severe complications during hospitalisation were more frequent in the AMIS-arm, but considering complications related to surgery, no difference was observed, with 35% of LAT and 39% of AMIS patients suffering no surgery-related complications. Low-grade complications treatable with common medications, like antiemetics, analgesics, etc., were experienced by 52% and 47% of patients, respectively. Six percent of patients from each group needed transfusions or antibiotics, while 7% from the LAT-arm and 8% from the AMIS-arm needed surgical treatment for surgery-associated complications, mainly infection. Overall complications were similarly distributed between the groups (see Additional file 13: Figure S5 for details).

None of the differences in secondary outcomes reached significance, even after adjusting for baseline differences in age or pfFIM status.

### Subgroup analyses

**Fig. 5:**
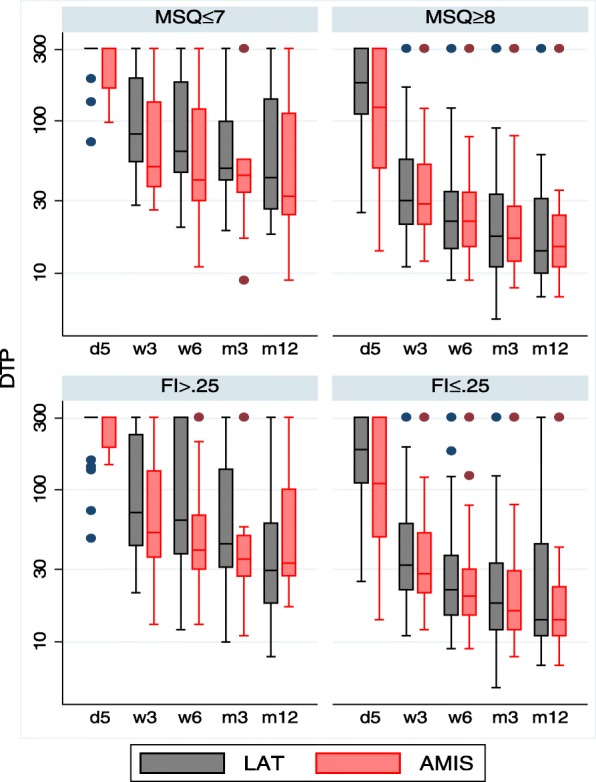
The distribution of duration of TUG performance (DTP) at day 5 and at all follow-up visits stratified by treatment arm and by normal and abnormal MSQ-values (upper panel) or by frailty index (lower panel). The figure illustrates larger differences in TUG duration for patients with low abnormal MSQ values or with high frailty index, respectively, especially at early time-points. Note that the y-axis uses a logarithmic scale

Figure 5 visualises the distribution of the variable “duration of TUG performance” by treatment arm after stratification by MSQ score. There were distinct differences favouring AMIS in patients with MSQ scores ≤7 (covering 26% of the population). However, there was nearly no difference in patients with MSQ scores ≥8, which was similar to the more pronounced effect in low-performing patients according to the pfFIM status observed above. Indeed, pfFIM status and MSQ score were highly correlated (*r* = 0.72). When using the frailty index of Arjunana et al. and a cut-off point of 0.25, the highly frail group included 26% of all patients, and we observed a similar pattern (Fig. 5). The frailty index was negatively correlated with pfFIM (*r* = − 0.90) and MSQ (*r* = − 0.63).

## Discussion

The data presented here support the conclusion that the muscle-sparing AMIS approach to the hip joint for HA can be beneficial for an early return to function and performance in ADLs, especially in potentially frail elderly patients, even if statistical superiority cannot be demonstrated. This corresponds to the results after THA, both considering the perceived benefit as well as the persistent lack of scientific evidence [22–27]. The dominance of differences at early time points between AMIS and traditional approaches might have a more significant impact in elderly patients with potentially reduced physical reserve because, unlike after THA, differences persisted over the first postoperative year in the population in our dataset. Renken et al. [31] reported similar results after HA for FNFs in 60 randomised elderly patients with better mobility and functioning in ADL at early time points after an AMIS approach compared to a Watson Jones approach, while other authors did not find differences [54–56] and concluded that there is an influence of surgeon proficiency, rather than the actual approach [55].

We observed a persistent influence of the treatment on function and ADL. This effect decreased over time, but this may be due to ceiling/floor effects, as many patients reach normal values for TUG performance or FIM scores. It might be assumed that the rather early occurrence and – in our dataset – persistence of outcome differences was related to the impact of hardly reversible perioperative deconditioning due to persistent pain or immobilisation, which has been associated with the traditional approaches. This tendency could also be an explanation for the more pronounced benefit of AMIS in patients with a higher risk of frailty, i.e. more frailty associated characteristics and potentially less reserve, to compensate for postoperative muscular hypotrophy.

This association points towards a methodological dilemma and might explain the current lack of high-level evidence for the superiority of minimally invasive surgery in the treatment of FNFs. There are few recently published RCTs analysing the benefit of minimally invasive surgery in patients with FNFs [31, 54–57]. The analysed patient populations tend to be relatively small, and most trials exclude cognitively impaired patients, who make up a large proportion of the affected elderly population. The heterogeneity of the population in principle complicates the generation of valid generalisable data.

In addition, the aspects of old age and cognitive impairment present a conceptual difficulty in RCTs. Cognitively impaired patients or patients diagnosed with dementia are typically excluded from interventional trials given the complexity of legitimate inclusion as well as the potential difficulty of meaningful data acquisition [58–60]. In our institution, approximately one-third of patients presenting with an FNF suffer from dementia. The rate of more subtle cognitive impairment is significantly higher. In these patients, an early return to their previous residential status and function is especially important [61]. Our data show a strong correlation between surrogate markers for frailty like FIM and MSQ as measure of cognitive impairment. There is also a marked correlation with the calculated frailty index comprising FIM, medication and comorbidities that has been reported as predictor of mortality, 30-day residence and length of inpatient stay. All of these measures predicted a benefit of AMIS over LAT as an approach for HA, which was specifically pronounced in patients with MSQ scores ≤7, i.e., in the presence of cognitive impairment.

In a literature review on minimally invasive THA, Jung et al. [62] reported consistently lower levels of pain and use of analgesics. The same effect has been documented after HA for FNFs in retrospective and prospective analyses [30, 31, 63], and this effect was also present in our data set, with patients in the AMIS-arm on average suffering lower levels of pain. However, the effect did not reach significance, unlike in previous studies [31, 63], and must be interpreted with caution since the agreement between formal ratings and verbal expressions of pain or discomfort seems to decrease with advancing age [64], and the reliability with cognitive impairment might be additionally limited.

Another aspect reported as an advantage after minimally invasive surgery is lower blood loss. We documented intraoperative blood loss as well as haemoglobin levels at different postoperative time points. We found, however, that these parameters were not reliable (or consistent) in our data set due to lack of standardised documentation of the intraoperative blood loss and individualised fluid management, as reported by other authors [31]. As a surrogate marker, the need for transfusion was evaluated, which is handled uniformly at our institution. We observed a higher need for transfusion within the first 72 h for patients treated with LAT compared to patients after AMIS as reported after HA via the AMIS approach. This is an important aspect in a comorbid patient population, since patients are not only at high risk of fluid overload and cardiac complications as a consequence of blood transfusion but also at a high risk of complications from anaemia such as delirium, fatigue and prolonged immobilisation. The above-mentioned RCTs on the subject report conflicting results in this aspect.

We should finally note that we observed a non-significant increase in mortality under AMIS, corresponding to 8 more deaths in 100 patients, without being able to give a clear explanation for the result. Further investigations in larger populations are necessary to clarify the question of a true difference in mortality.

### Strengths and limitations

The strength of the current trial is its RCT design with a relatively high number of patients, including patients with cognitive deficits, which reduces the potential for selection bias. The early assessment of TUG as a functional parameter for the primary outcome makes the current results predictive for performance in ADLs and for preservation of independence [34, 35], which are highly patient-relevant endpoints. However, we did not include instruments sensitive enough to catch such long-term differences. An additional strength is the large number of participating surgeons and their allocation by independent administrative procedures, resulting in a highly balanced allocation and avoiding a bias in treatment effect estimation due to experience-based selection of surgeons. A uniform level of surgical quality could be ascertained by the presence of a senior expert.

We included in the analysis of the primary outcome 144 patients, nearly reaching the intended sample size of 150 as planned by the power analysis. We also observed a difference in median duration of TUG performance of 6 s (LAT: 41 s, AMIS: 35 s), corresponding to the assumed effect in the sample size calculation and hitting the MCID reported for the TUG [36]. The failure to reach significance may be due to our decision to include in the analysis patients who failed to perform the TUG successfully with a duration of 300 s, which is in contrast to other studies ignoring these patients. In this way, we reduced the potential for bias, but we also increased the variability and hence reduced the power. Indeed, exclusion of these patients leads to a smaller treatment effect estimate of − 16.8%, with a smaller *p*-value of 0.089.

The current trial is limited by the high rate of screening failures, which is typical in prospective RCTs in elderly patients [59]. Furthermore, a certain loss to follow-up and failure to perform the TUG is problematic. However, sensitivity analyses suggest that the main findings are robust against different ways of handling missing values. The patient numbers in the two treatment arms show a distinct – but insignificant – difference, reflecting the lack of block randomisation. We also have to note that the FIM is constructed as an observational tool for nursing home staff with close contact to patients, but it was used in our trial by study nurses relying on patient or proxy narratives. We did not perform a specific assessment of the pre-fracture frailty in this trial, and hence we were forced to use surrogate measures to investigate a dependence of the treatment effect on frailty.

## Conclusion

FNFs in elderly patients are frequent, and given demographic developments, their socio-economic impact will increase in coming decades. Every step contributing to a better outcome in these patients is beneficial for the individual patient and for society. In this regard, the present trial – despite failing to reach a significant difference in the primary outcome – adds to a growing body of evidence. These results support the implementation of a specifically designed treatment regimen for fragility fractures, taking into account the complexity of the heterogeneous patient population known as “the elderly”.

## Additional files

Additional file 1: Definition of Frailty Index. Description of the approach for the quantification of frailty. (DOCX 13 kb)

Additional file 2: Statistical approach for analysing the primary outcome. Description of the analytical approach for the statistical analysis of the primary outcome with display of the primary outcome with and without a logarithmic transformation in Additional file 3: Figure S1. (DOCX 13 kb)

Additional file 3: **Figure S1.** The distribution of the duration of TUG performance (DTP) at 3 weeks in the two treatment arms with and without a logarithmic transformation. Comparing the upper and lower part, we can observe that the logarithmic transformation implies a less skewed distribution of the primary outcome in both arms of the study. (DOCX 20 kb)

Additional file 4: **Table S1.** Participation rates at follow-up visits and percentages of patients performing TUG or having assessed FIM, respectively. The percentages refer to the number of patients attending the visit. (DOCX 12 kb)

Additional file 5: Predictors for drop out. Description of the analytic strategy for the evaluation of the potential influence of patient characteristics on the availability of outcome parameters with display of the results in Additional file 6: Table S2. (DOCX 13 kb)

Additional file 6: **Table S2.** Association of patient characteristics and availability of the primary outcome. Association of patient characteristics and previous measurements with non-attendance at follow-up visits, non-availability of DTP or non-performance of TUG when attending. The observed ORs in each treatment arm, the *p*-value of an overall effect of the variable (p1), and the *p*-value of a test for equality across the two arms (p2) are given. For continuous variables, the OR refers to changing this variable by one standard deviation. (DOCX 13 kb)

Additional file 7: **Table S3.** Distribution of baseline characteristics in the two treatment groups among patients entering the analysis of the primary endpoint. The table illustrates larger differences in baseline characteristics for patients with complete data for the analysis of the primary outcome compared to Table 3 showing the baseline characteristics of all patients. (DOCX 15 kb)

Additional file 8: Sensitivity analyses. Description of the analytic strategy for the evaluation of the potential influence of different ways to handle missing values on the effect estimates with display of the results in Additional file 9: Table S4. (DOCX 13 kb)

Additional file 9: **Table S4.** Variation of treatment effect estimates, confidence intervals and *p*-values for the outcomes DTP and FIM at 3 weeks and at day 5 across six different approaches to handle missing values. (DOCX 13 kb)

Additional file 10: **Figure S2.** Distribution of FIM at all time-points in relation to treatment and prefecture FIM (prFIM). The lines refer to running medians based on the next 25 neighbours on both sides of an observation. The area of each point is proportional to the number of observations with the specific combination of FIM and prFIM value. With increasing time postoperative an increasing number of patients can reach their pre-fracture level of independence. The advantage of AMIS in comparison to FIM is more pronounced in patients with low prefracture FIM values. (DOCX 142 kb)

Additional file 11: **Figure S3.** The individual trajectories of DTP and FIM for all patients. With respect to DTP the typical pattern is a continuous improvement over time. However, in both arms a few patients experience a sudden deterioration. With respect to FIM the typical pattern is a distinct deterioration due to the fracture/surgery and a continuous improvement afterwards. However, in both treatment arms some patients get stuck in the recovering process. (DOCX 39 kb)

Additional file 12: **Figure S4**. Distribution of the VAS pain scores at all time-points in relation to treatment and pre-fracture FIM status (prFIM). The lines refer to running means based on the next 25 neighbours on both sides of an observation. The area of each point is proportional to the number of observations with the specific combination of VAS and prFIM value. We observe higher mean VAS values in the LAT arm compared to the AMIS arm in partucular for patients with low pre-fractureFIM values at week 3, week6 and month 3. (DOCX 321 kb)

Additional file 13: **Figure S5.** Distribution of in-hospital complications according to the Clavien-Dindo classification. In this context, only in-hospital complications are presented as the rate of later complications was low and might reflect an underreporting especially of infections treated by the GP in this patient population. (DOCX 21 kb)

## Abbreviations

ADL: Activities of daily living
AMIS: Anterior minimally invasive surgery
BMI: Body mass index
CAM: Confusion Assessment Method
DOS: Delirium Observation Screening scale
FI: Frailty index
FIM: Functional independence measure
FNF: Femoral neck fracture
HA: Hemiarthroplasty
LAT: Lateral transgluteal Hardinge approach
LOS: Length of (hospital) stay
MSQ: Mental status questionnaire
pfFIM: Pre-fracture functional independence measure
RCT: Randomised controlled trial
SAE: Serious adverse events
THA: Total hip arthroplasty
TUG: “Timed up and go”-test
VAS: Visual analogue scale
