# Supplementary material for: Minimally invasive anterior muscle-sparing versus a transgluteal approach for hemiarthroplasty in femoral neck fractures-a prospective randomised controlled trial including 190 elderly patients
Source: BMC Geriatr. 2018 Sep 21;18:222. doi: 10.1186/s12877-018-0898-9 (PMC6151034; doi:10.1186/s12877-018-0898-9)
Supplement: Supplementary file 1 — Definition of Frailty Index. Description of the approach for the quantification of frailty. (DOCX 13 kb) [file 12877_2018_898_MOESM1_ESM.docx]

To obtain a quantification of the degree of frailty, we made use of an frailty index (FI) recently suggested by Arjunan et al (2018 as referenced in the main document), which is based on the FIM, the Charlson Index and the number of medications. All these quantities were measured also in this study. However, we only documented the overall FIM, and not the results for the single items. Hence we were not able to apply exactly the procedure of Arjunan et al, as they suggest recoding the single FIM items mapping the values 6 and 7 to 0, 2 to 5 to 0.5, and 1 to 1. We replaced this step by just rescaling the original FIM item scale (from 7 down to 1) to the interval from 0 up to 1. Consequently, our formula for the FI was

(18-(FIM-18)/6 + Charlson Index + Medication score)/36

with the medication score mapping the intervals 0-4, 5-9, 10-14, 15-19 and >=20 of the number of medications at hospitalization to 0, 1, 2, 3, and 4, exactly as suggested by Arjunan et al.
