# Supplementary material for: Minimally invasive anterior muscle-sparing versus a transgluteal approach for hemiarthroplasty in femoral neck fractures-a prospective randomised controlled trial including 190 elderly patients
Source: BMC Geriatr. 2018 Sep 21;18:222. doi: 10.1186/s12877-018-0898-9 (PMC6151034; doi:10.1186/s12877-018-0898-9)
Supplement: Supplementary file 2 — Statistical approach for analysing the primary outcome. Description of the analytical approach for the statistical analysis of the primary outcome with display of the primary outcome with and without a logarithmic transformation in Additional file 3: Figure S1. (DOCX 13 kb) [file 12877_2018_898_MOESM2_ESM.docx]

A right-skewed distribution was expected (and actually observed) in our data for the duration of the TUG performance (DTP). To take this into account a log-transformation was applied, which indeed led to a less skewed distribution in the two treatment arms (Supplementary Figure 1), allowing a more robust analysis. The study protocol suggested adjusting for baseline functional ability considering the three candidate variables age, BMI and pfFMI, which were supposed to be selected according to their correlation with the primary outcome. We observed Pearson correlations of 0.25, -0.04 and -0.40, respectively for age and pfFIM and decided to adjust accordingly for both. Applying a regression model with the covariates arm, age and pfFIM to the log-transformed values, we obtain an estimate of the treatment effect corresponding to the difference in expected DTP between the two arms when comparing two individuals not differing in age and pfFIM. Assuming that the conditional distribution of DTP is symmetrical we can interpret this also as the difference in median DTP. Since differences on a log-scale can be back-transformed to a ratio on the original scale, we can thus obtain an estimate for the ratio *r* between the medians. (This does not hold true for the expectations, which is the main reason for considering medians instead of means.) The transformation *100*(r-1*) results then in the percentage-difference between the arm-specific medians.

This approach has been specified in the study protocol; except of that there an adjustment for only one of the candidate variables was suggested. Since we observed correlations for two candidates, we decided to adjust for both.
