# Supplementary material for: Minimally invasive anterior muscle-sparing versus a transgluteal approach for hemiarthroplasty in femoral neck fractures-a prospective randomised controlled trial including 190 elderly patients
Source: BMC Geriatr. 2018 Sep 21;18:222. doi: 10.1186/s12877-018-0898-9 (PMC6151034; doi:10.1186/s12877-018-0898-9)
Supplement: Supplementary file 4 — Table S1. Participation rates at follow-up visits and percentages of patients performing TUG or having assessed FIM, respectively. The percentages refer to the number of patients attending the visit. (DOCX 12 kb) [file 12877_2018_898_MOESM4_ESM.docx]

|  | **Attendance to visit** | | **TUG performed** | | **FIM assessed** | |
| --- | --- | --- | --- | --- | --- | --- |
|  | LAT | AMIS | LAT | AMIS | LAT | AMIS |
|  | n % | n % | n % | n % | n % | n % |
| baseline | 99 100.0% | 82 100.0% |  |  | 93 93.9% | 75 91.5% |
| day 5 | 95 96.0% | 82 100.0% | 85 89.5% | 63 76.8% | 95 100.0% | 76 92.7% |
| week 3 | 90 90.9% | 73 89.0% | 80 88.9% | 66 90.4% | 90 100.0% | 73 100.0% |
| week 6 | 85 85.9% | 66 80.5% | 80 94.1% | 63 95.5% | 85 100.0% | 66 100.0% |
| month 3 | 78 78.8% | 60 73.2% | 76 97.4% | 58 96.7% | 78 100.0% | 60 100.0% |
| month 12 | 59 59.6% | 47 57.3% | 55 93.2% | 42 89.4% | 59 100.0% | 47 100.0% |
