# Supplementary material for: Minimally invasive anterior muscle-sparing versus a transgluteal approach for hemiarthroplasty in femoral neck fractures-a prospective randomised controlled trial including 190 elderly patients
Source: BMC Geriatr. 2018 Sep 21;18:222. doi: 10.1186/s12877-018-0898-9 (PMC6151034; doi:10.1186/s12877-018-0898-9)
Supplement: Supplementary file 5 — Predictors for drop out. Description of the analytic strategy for the evaluation of the potential influence of patient characteristics on the availability of outcome parameters with display of the results in Additional file 6: Table S2. (DOCX 13 kb) [file 12877_2018_898_MOESM5_ESM.docx]

*Statistical methods*

To identify predictors for non-attendance of a follow-up visit, missing DTP measurements or for non-performance of TUG if attending a visit, we considered associations to selected patient characteristics. In order to obtain reliable estimates, we pooled the data from all time points including all patients at risk. All patients attending the previous follow-up time point were at risk for not attending the next one, all patients having performed TUG at the previous follow-up time point were at risk of having no DTP measurement at the next time point, and finally all patients who attended were at risk for non-performance of TUG. The association was described by odds ratios from a logistic regression adjusting for the effect of time with robust standard errors to take the clustering within the patient-population into account. Similarity of the association between the two arms was assessed by estimating arm-specific odds ratios and significance testing for a difference.

*Results*

As mentioned above, we have a non-negligible rate of drop-outs and non-performance of TUG. Table S2 depicts the association of these events with some patient characteristics. We can observe a distinct trend for old patients, patients not living at home on admission, patients with low pfFIM, with low MSQ and those with low FIM or high DTP at the previous follow-up to attend less frequently. With regards to missing the DTP measurement, also the associations with dementia and walking aid become significant. In case of attendance, the associations with non-performance of TUG were less pronounced. No differences in the degree of associations between the treatment arms could be found, except for the use of walking aids with a more pronounced association in the LAT arm. This may reflect that patients already initially using a walking aid are rather able or willing to mobilize and actively participate in assessments after treatment in the AMIS arm. No significant gender differences could be observed.
