# Supplementary material for: Minimally invasive anterior muscle-sparing versus a transgluteal approach for hemiarthroplasty in femoral neck fractures-a prospective randomised controlled trial including 190 elderly patients
Source: BMC Geriatr. 2018 Sep 21;18:222. doi: 10.1186/s12877-018-0898-9 (PMC6151034; doi:10.1186/s12877-018-0898-9)
Supplement: Supplementary file 6 — Table S2. Association of patient characteristics and availability of the primary outcome. Association of patient characteristics and previous measurements with non-attendance at follow-up visits, non-availability of DTP or non-performance of TUG when attending. The observed ORs in each treatment arm, the p-value of an overall effect of the variable (p1), and the p-value of a test for equality across the two arms (p2) are given. For continuous variables, the OR refers to changing this variable by one standard deviation. (DOCX 13 kb) [file 12877_2018_898_MOESM6_ESM.docx]

|  | Not attended | | | | No DTP measurement  available | | | | No TUG performed | | | |
| --- | --- | --- | --- | --- | --- | --- | --- | --- | --- | --- | --- | --- |
|  | LAT | AMIS | p_1_ | p_2_ | LAT | AMIS | p_1_ | p_2_ | LAT | AMIS | p_1_ | p_2_ |
| Age | 2.3 | 1.8 | 0.036 | 0.723 | 2.9 | 1.8 | <.001 | 0.301 | 4.4 | 1.2 | 0.047 | 0.088 |
| pfFIM | 0.4 | 0.3 | <.001 | 0.617 | 0.5 | 0.7 | 0.007 | 0.555 | 0.8 | 1.7 | 0.737 | 0.353 |
| Dementia | 1.2 | 1.8 | 0.124 | 0.444 | 1.2 | 1.5 | 0.036 | 0.511 | 0.5 | 0.8 | 0.291 | 0.502 |
| Gender | 1.0 | 0.9 | 0.880 | 0.902 | 1.1 | 0.7 | 0.742 | 0.238 | 2.0 | 0.7 | 0.477 | 0.103 |
| Living at home | 0.6 | 0.5 | 0.005 | 0.692 | 0.9 | 0.6 | 0.009 | 0.182 | 1.1 | 1.1 | 0.919 | 0.895 |
| MSQ | 0.5 | 0.2 | <.001 | 0.133 | 0.5 | 0.3 | <.001 | 0.273 | 0.7 | 1.1 | 0.771 | 0.592 |
| Walking aid | 1.9 | 1.1 | 0.103 | 0.295 | 2.0 | 0.9 | 0.044 | 0.011 | 2.2 | 0.8 | 0.252 | 0.045 |
| Prevalue of FIM | 0.2 | 0.2 | <.001 | 0.827 | 0.2 | 0.3 | <.001 | 0.294 | 0.2 | 1.2 | 0.121 | 0.071 |
| Prevalue of DTP | 7.0 | 3.3 | <.001 | 0.536 | 7.0 | 2.4 | <.001 | 0.233 | 6.4 | 1.2 | 0.073 | 0.236 |
