# Supplementary material for: Minimally invasive anterior muscle-sparing versus a transgluteal approach for hemiarthroplasty in femoral neck fractures-a prospective randomised controlled trial including 190 elderly patients
Source: BMC Geriatr. 2018 Sep 21;18:222. doi: 10.1186/s12877-018-0898-9 (PMC6151034; doi:10.1186/s12877-018-0898-9)
Supplement: Supplementary file 7 — Table S3. Distribution of baseline characteristics in the two treatment groups among patients entering the analysis of the primary endpoint. The table illustrates larger differences in baseline characteristics for patients with complete data for the analysis of the primary outcome compared to Table 3 showing the baseline characteristics of all patients. (DOCX 15 kb) [file 12877_2018_898_MOESM7_ESM.docx]

|  |  | LAT | AMIS |  |
| --- | --- | --- | --- | --- |
| Age | n | 79 | 65 | p=0.548 |
|  | mean (sd) | 83.2 (6.6) | 83.8 (6.8) |  |
|  | median (10%,90%) | 84.0 (74.0,90.0) | 86.0 (73.0,91.0) |  |
| Gender | male | 28/79 35.4% | 14/65 21.5% | p=0.069 |
|  | female | 51/79 64.6% | 51/65 78.5% |  |
| Body Mass Index | n | 79 | 59 | p=0.916 |
|  | mean (sd) | 23.5 (4.8) | 23.8 (4.7) |  |
|  | median (10%,90%) | 24.0 (18.0,28.0) | 23.0 (18.0,29.0) |  |
| Residential status | own home | 49/79 62.0% | 31/65 47.7% | p=0.170 |
|  | own home supported | 8/79 10.1% | 10/65 15.4% |  |
|  | assisted living | 4/79 5.1% | 7/65 10.8% |  |
|  | nursing home | 15/79 19.0% | 16/65 24.6% |  |
|  | other | 3/79 3.8% | 1/65 1.5% |  |
| Walking aid | yes | 33/79 41.8% | 42/65 64.6% | p=0.007 |
| Dementia | yes | 14/79 17.7% | 19/65 29.2% | p=0.103 |
| MSQ | n | 74 | 62 | p=0.180 |
|  | mean (sd) | 8.1 (2.8) | 7.6 (3.1) |  |
|  | median (10%,90%) | 9.0 (4.0,10.0) | 9.0 (2.0,10.0) |  |
| pfFIM | n | 79 | 65 | p=0.271 |
|  | mean (sd) | 108.3 (26.0) | 105.7 (25.6) |  |
|  | median (10%,90%) | 123.0 (63.0,126.0) | 117.0 (58.0,126.0) |  |
| Number of medications | n | 79 | 65 | p=0.412 |
|  | mean (sd) | 7.1 (4.6) | 6.4 (4.3) |  |
|  | median (10%,90%) | 6.0 (1.0,14.0) | 6.0 (1.0,12.0) |  |
| Charlson Comorbidity Score | n | 79 | 64 | p=0.276 |
|  | mean (sd) | 2.2 (2.1) | 2.6 (2.3) |  |
|  | median (10%,90%) | 2.0 (0.0,5.0) | 2.0 (0.0,6.0) |  |
| Frailty Index | n | 79 | 64 | p=0.427 |
|  | mean (sd) | 0.17 (0.16) | 0.19 (0.16) |  |
|  | median (10%,90%) | 0.11 (0.01,40) | 0.15 (0.03,0.44) |  |
| ASA score | 2 | 27/79 34.2% | 19/65 29.2% | p=0.377 |
|  | 3 | 50/79 63.3% | 42/65 64.6% |  |
|  | 4 | 2/79 2.5% | 4/65 6.2% |  |
| Time until surgery (hrs) | n | 79 | 65 | p=0.797 |
|  | mean (sd) | 26.7 (19.1) | 25.8 (14.5) |  |
|  | median (10%,90%) | 22.0 (5.0,52.0) | 25.0 (7.0,44.0) |  |
| Haemoglobin | n | 79 | 65 | p=0.880 |
|  | mean (sd) | 130.6 (15.8) | 131.2 (15.6) |  |
|  | median (10%,90%) | 134.0 (110.0,149.0) | 131.0 (111.0,150.0) |  |
| Creatinine | n | 79 | 64 | p=0.306 |
|  | mean (sd) | 20.1 (10.1) | 18.2 (8.1) |  |
|  | median (10%,90%) | 18.0 (10.0,32.0) | 16.5 (10.0,29.0) |  |
| Albumin | n | 79 | 65 | p=0.287 |
|  | mean (sd) | 33.9 (7.5) | 34.0 (4.8) |  |
|  | median (10%,90%) | 35.0 (28.0,40.0) | 34.0 (29.0,39.0) |  |
| CRP | n | 79 | 65 | p=0.341 |
|  | mean (sd) | 14.5 (29.0) | 16.6 (25.1) |  |
|  | median (10%,90%) | 4.0 (1.0,56.0) | 6.0 (0.0,43.0) |  |
| Leukocytes | n | 79 | 65 | p=0.813 |
|  | mean (sd) | 10.5 (3.7) | 10.5 (3.4) |  |
|  | median (10%,90%) | 10.0 (6.0,15.0) | 10.0 (7.0,16.0) |  |
