# Supplementary material for: Minimally invasive anterior muscle-sparing versus a transgluteal approach for hemiarthroplasty in femoral neck fractures-a prospective randomised controlled trial including 190 elderly patients
Source: BMC Geriatr. 2018 Sep 21;18:222. doi: 10.1186/s12877-018-0898-9 (PMC6151034; doi:10.1186/s12877-018-0898-9)
Supplement: Supplementary file 8 — Sensitivity analyses. Description of the analytic strategy for the evaluation of the potential influence of different ways to handle missing values on the effect estimates with display of the results in Additional file 9: Table S4. (DOCX 13 kb) [file 12877_2018_898_MOESM8_ESM.docx]

*Statistical methods*

We used multiple imputation to perform sensitivity analyses with respect to handling drop-outs focussing on the effect estimates with FIM or DTP as outcomes after 5 days and 3 weeks. After imputing fixed values in the FIM and DTP variables for those who died prior to the intended measurement, we generated multiple imputations based on chained equations in combination with predictive mean matching. We included gender, age and treatment arm as completely observed variables, living at home, the use of walking aids and the pfFIM as partially observed baseline variables, FIM and the logarithm of DTP at day 5 and after 3 weeks in the multiple imputation approach. The prediction models included always the square of each continuous covariate.

Results

The influence of different ways to handle missing values in the DTP and FIM variables on the effect estimates is investigated in Supplementary Table S4. We considered 4 different approaches: Using only the patients with complete information on the outcome and the pfFIM (complete case analysis – CC – as applied in Supplementary Table S3), imputing the values 300 for DTP and the minimally possible value 17 for FIM, respectively, if a patient died prior to the visit, multiple imputation under the assumption that the occurrence of missing values is not informative (Missing at random – MAR) and multiple imputation under the assumption that unobserved DTP-values are 25% higher and unobserved FIM-values are 20% lower than under MAR (NonMAR). For MAR and NonMAR we consider two variants, one assuming a joint model over both arms and one assuming arm-specific models. We observe that the effect estimates, confidence intervals and p-values are rather stable if we consider the primary outcome and tend to be more pronounced than in the CC analysis. In contrast, the treatment effect may become less pronounced when considering DTP at day 5 or FIM at one of the two time points. However, overall we can conclude that effect estimates are not very sensitive to the choice of how to handle missing values.
