# Supplementary material for: Minimally invasive anterior muscle-sparing versus a transgluteal approach for hemiarthroplasty in femoral neck fractures-a prospective randomised controlled trial including 190 elderly patients
Source: BMC Geriatr. 2018 Sep 21;18:222. doi: 10.1186/s12877-018-0898-9 (PMC6151034; doi:10.1186/s12877-018-0898-9)
Supplement: Supplementary file 9 — Table S4. Variation of treatment effect estimates, confidence intervals and p-values for the outcomes DTP and FIM at 3 weeks and at day 5 across six different approaches to handle missing values. (DOCX 13 kb) [file 12877_2018_898_MOESM9_ESM.docx]

|  | | effect | 95%-CI | p-value |  | | | effect | 95%-CI | p-value |
| --- | --- | --- | --- | --- | --- | --- | --- | --- | --- | --- |
| DTP week 3 | | | | | FIM week 3 | | | | | |
|  | CC | -21.5 | [-41.2,4.7] | 0.101 |  | CC | | 6.7 | [0.5,12.8] | 0.037 |
|  | Impute if dead | -23.5 | [-43.0,2.7] | 0.076 |  | Impute if dead | | 6.7 | [0.0,13.4] | 0.050 |
|  | MAR-joint | -22.9 | [-41.2,1.3] | 0.063 |  | MAR-joint | | 6.6 | [-0.1,13.4] | 0.054 |
|  | MAR-arm | -21.5 | [-40.8,4.2] | 0.095 |  | MAR-arm | | 5.8 | [-1.0,12.6] | 0.094 |
|  | NonMAR-joint | -24.7 | [-44.7,2.3] | 0.071 |  | NonMAR-joint | | 6.1 | [-0.3,12.4] | 0.064 |
|  | NonMAR-arm | -23.5 | [-44.3,5.1] | 0.100 |  | NonMAR-arm | | 5.5 | [-1.0,11.9] | 0.099 |
| DTP day 5 | | | | | FIM day 5 | | | | | |
|  | CC | -25.3 | [-41.0,-5.4] | 0.017 |  | CC | 4.0 | | [-1.7,9.7] | 0.169 |
|  | Impute if dead | -25.3 | [-41.0,-5.4] | 0.017 |  | Impute if dead | 4.0 | | [-1.7,9.7] | 0.169 |
|  | MAR-joint | -23.9 | [-38.1,-6.4] | 0.010 |  | MAR-joint | 4.3 | | [-1.4,9.9] | 0.138 |
|  | MAR-arm | -23.3 | [-37.6,-5.7] | 0.013 |  | MAR-arm | 3.5 | | [-2,2,9.2] | 0.227 |
|  | NonMAR-joint | -17.9 | [-38.8,6.6] | 0.140 |  | NonMAR-joint | 4.0 | | [-1.4,9.4] | 0.146 |
|  | NonMAR-arm | -17.2 | [-36.2,7.4] | 0.156 |  | NonMAR-arm | 3.4 | | [-2.0,8.8] | 0.218 |
