# Supplementary figures and images for: Minimally invasive anterior muscle-sparing versus a transgluteal approach for hemiarthroplasty in femoral neck fractures-a prospective randomised controlled trial including 190 elderly patients
Source: BMC Geriatr. 2018 Sep 21;18:222. doi: 10.1186/s12877-018-0898-9 (PMC6151034; doi:10.1186/s12877-018-0898-9)

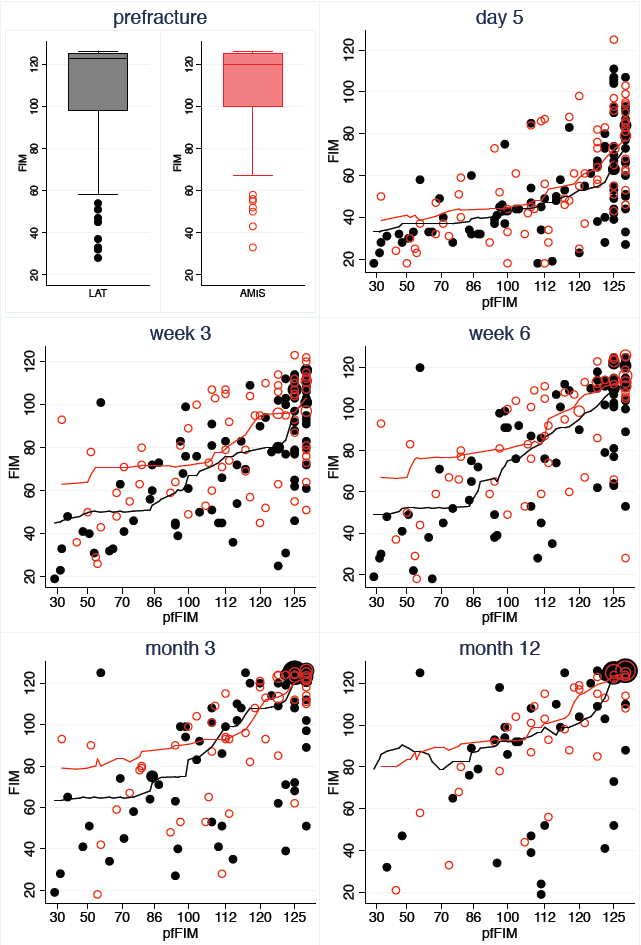

Supplement: Supplementary file 10 — Figure S2. Distribution of FIM at all time-points in relation to treatment and prefecture FIM (prFIM). The lines refer to running medians based on the next 25 neighbours on both sides of an observation. The area of each point is proportional to the number of observations with the specific combination of FIM and prFIM value. With increasing time postoperative an increasing number of patients can reach their pre-fracture level of independence. The advantage of AMIS in comparison to FIM is more pronounced in patients with low prefracture FIM values. (DOCX 142 kb) [file 12877_2018_898_MOESM10_ESM.docx]

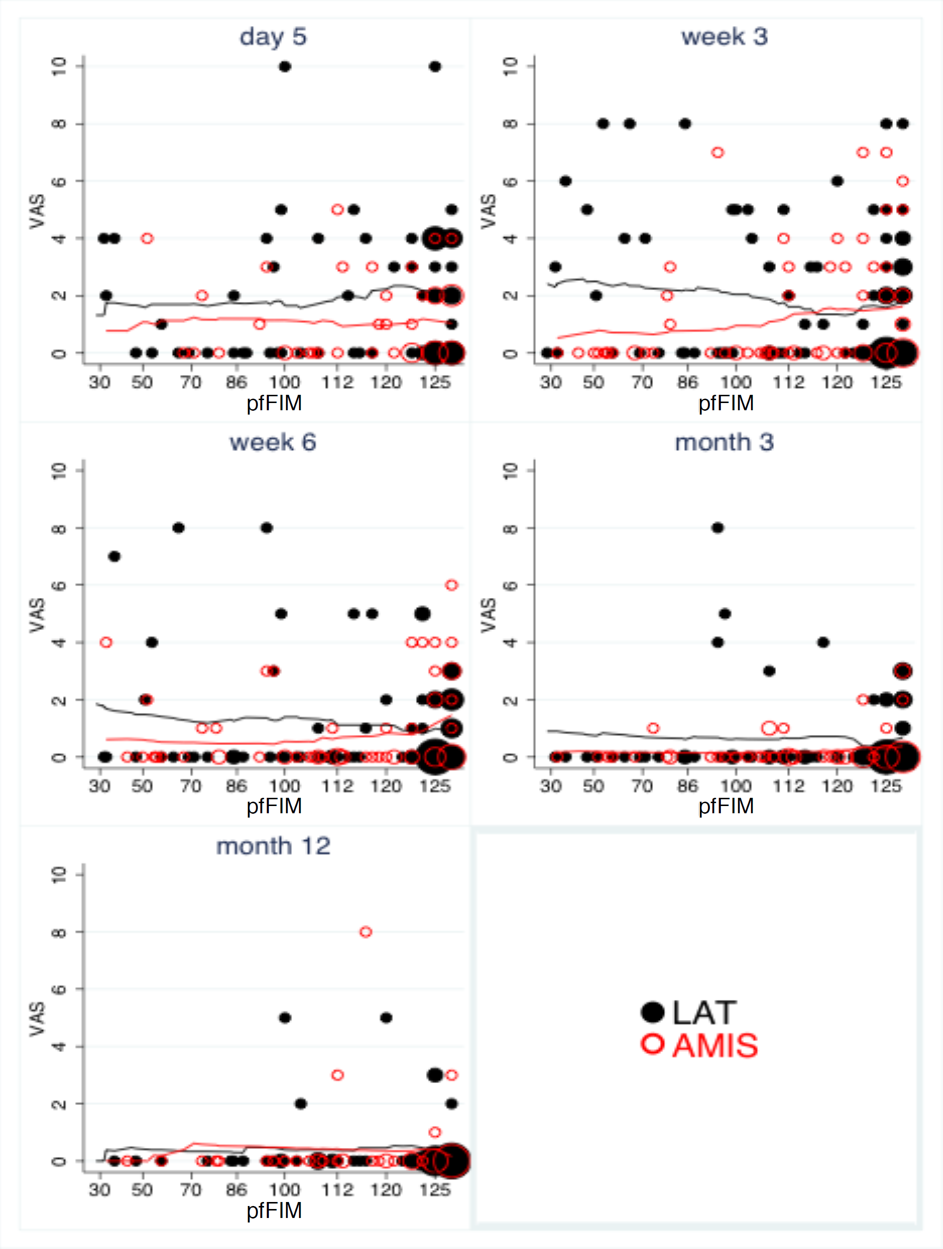

Supplement: Supplementary file 12 — Figure S4. Distribution of the VAS pain scores at all time-points in relation to treatment and pre-fracture FIM status (prFIM). The lines refer to running means based on the next 25 neighbours on both sides of an observation. The area of each point is proportional to the number of observations with the specific combination of VAS and prFIM value. We observe higher mean VAS values in the LAT arm compared to the AMIS arm in partucular for patients with low pre-fractureFIM values at week 3, week6 and month 3. (DOCX 321 kb) [file 12877_2018_898_MOESM12_ESM.docx]
