# Supplementary material for: Minimally invasive anterior muscle-sparing versus a transgluteal approach for hemiarthroplasty in femoral neck fractures-a prospective randomised controlled trial including 190 elderly patients
Source: BMC Geriatr. 2018 Sep 21;18:222. doi: 10.1186/s12877-018-0898-9 (PMC6151034; doi:10.1186/s12877-018-0898-9)
Supplement: Supplementary file 13 — Figure S5. Distribution of in-hospital complications according to the Clavien-Dindo classification. In this context, only in-hospital complications are presented as the rate of later complications was low and might reflect an underreporting especially of infections treated by the GP in this patient population. (DOCX 21 kb) [file 12877_2018_898_MOESM13_ESM.docx]

**Grade I** Any deviation from the normal postoperative course without the need for pharmacological treatment or surgical, endoscopic and radiological intervention. Allowed therapeutic regimens are: drugs as antiemetics, antipyretics, analgesics, diuretics and electrolytes and physiotherapy. This grade also includes wound infections opened at the bedside.

**Grade II** Requiring pharmacological treatment with drugs other than such allowed for grade I complications. Blood transfusions and total parenteral nutrition are also included

**Grade III** Requiring surgical, endoscopic or radiological intervention

**Grade IV**  Life- threatening complication (including central nervous system complications) requiring intensive-care-management

**Grade V** Death of a patient
