# Supplementary material for: Minimally invasive anterior muscle-sparing versus a transgluteal approach for hemiarthroplasty in femoral neck fractures-a prospective randomised controlled trial including 190 elderly patients
Source: BMC Geriatr. 2018 Sep 21;18:222. doi: 10.1186/s12877-018-0898-9 (PMC6151034; doi:10.1186/s12877-018-0898-9)
Supplement: Supplementary file 11 [file 12877_2018_898_MOESM11_ESM.docx]

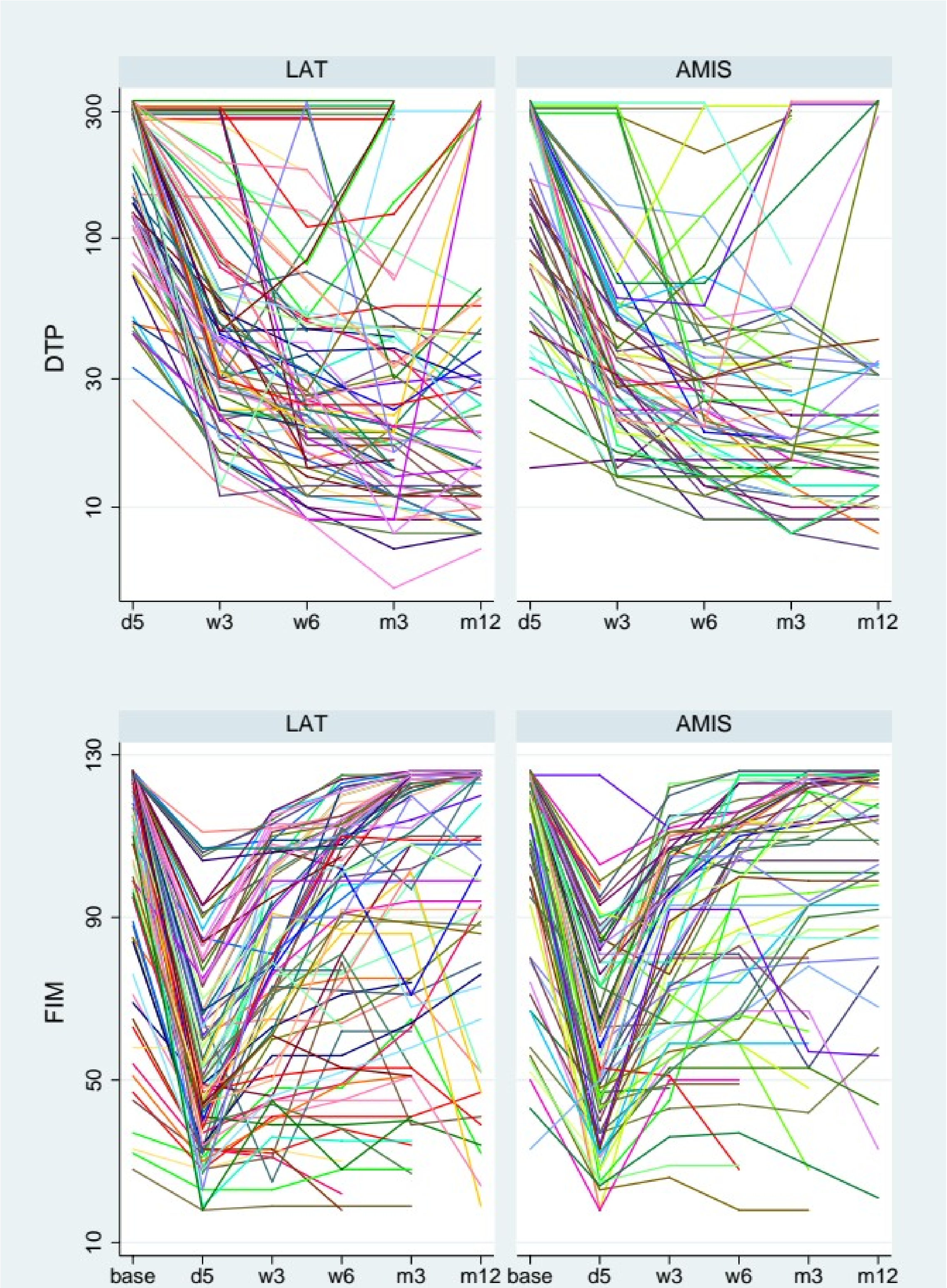

LAT
AMIS
DTP
300
100
30
10
d5
w3
w6
m3
m12
FIM
130
90
50
10
base
