# Supplementary material for: Minimally invasive anterior muscle-sparing versus a transgluteal approach for hemiarthroplasty in femoral neck fractures-a prospective randomised controlled trial including 190 elderly patients
Source: BMC Geriatr. 2018 Sep 21;18:222. doi: 10.1186/s12877-018-0898-9 (PMC6151034; doi:10.1186/s12877-018-0898-9)
Supplement: Supplementary file 12 [file 12877_2018_898_MOESM3_ESM.docx]

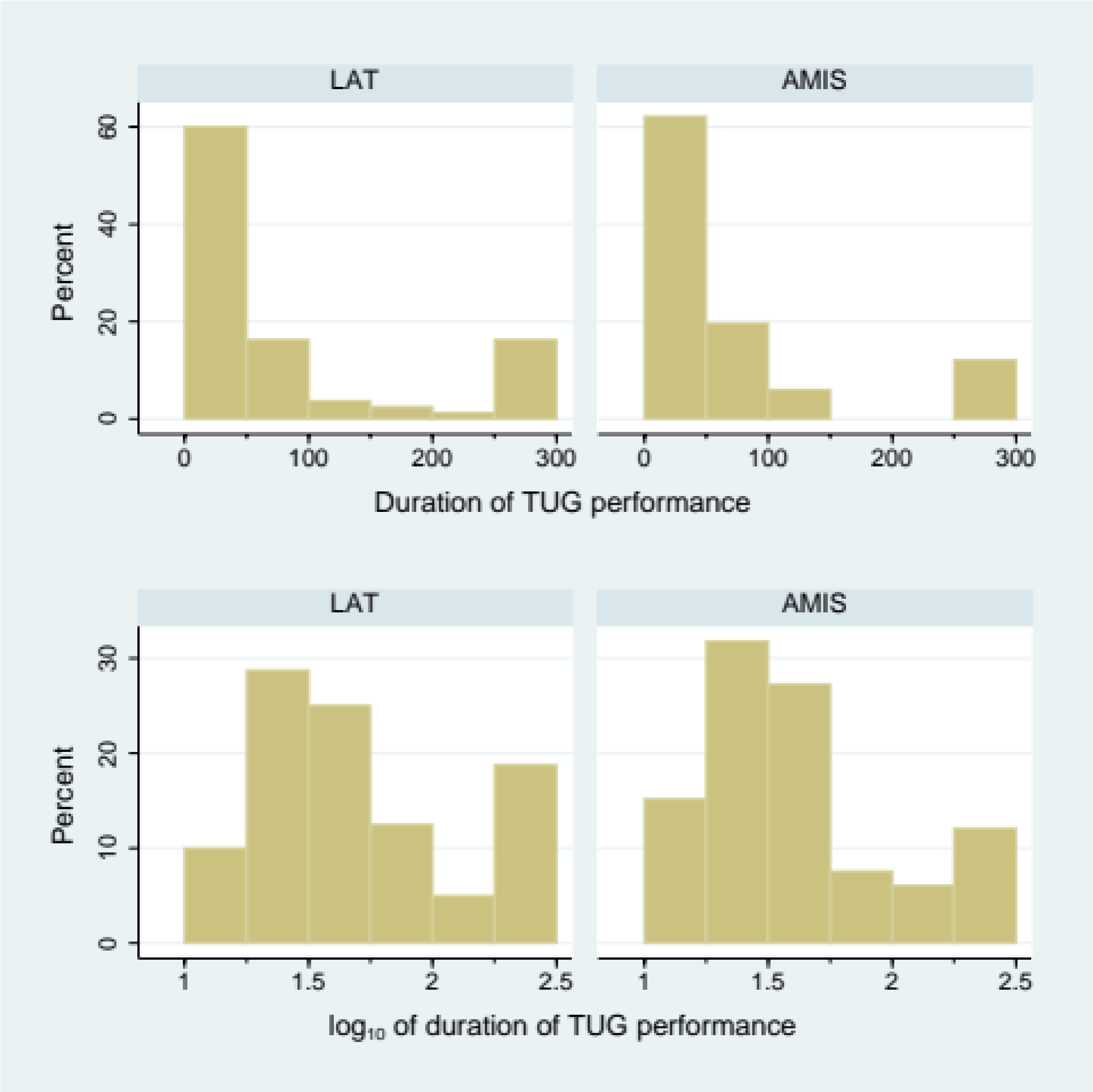
LAT
AMIS
Percent
0
20
40
60
0
100
200
300
Duration of TUG performance
LAT
AMIS
Percent
0
10
20
30
1
1.5
2
2.5
log10 of duration of TUG performance
